# Hats and Their Relation to Men

**Published:** 1885-07

**Authors:** Ausburn Towner


### HATS AND THEIR RELATION TO MEN.

AUSBURN TOWNER.

The head has always been esteemed the superior portion of the human person, and that which has protected or covered it, in all ages and in the various civilizations of the world, has been considered of the highest dignity and worth. In this hard prosaic age, there is little left to sentiment or mysticism, and unless we stop and reflect, we forget how much there is of both in our every-day life, and that we can hardly utter a sentence on no matter how commonplace a topic, that has not in it, words whose use comes to us through a strain of poetry or comparison and likeness. How can man go higher than his head where sit his reasoning powers, his controlling forces? How natural then that that which is above this, which covers and protects it should come to mean also the highest, the top, the uppermost, above which, nothing; as the "cap" of a mountain peak; "the cloud-capped" towers of the "Tempest"; the "cap" stone of a building or lofty monument, and in abstract things the consummation and completion of an undertaking, "to cap it all."

It is easily to be recollected with what power certain head coverings have appealed to the world and for how much they stand in history. The sacred and sorrowful crown of thorns; the turban of the prophet; the cap of Gesler; the white plume of Navarre and a certain plume of a later date not quite so successful, both these latter involving, of course, the notion of a cap or head-piece, for why the plume unless something to which to fasten it? All of these are but illustrations of the influence exerted in one way or another by those objects that attained a dignity by being placed originally on the head of some leader of men. And how better or more accurately could epochs, races, or periods be symbolized than by the head-gear prominent among them? As the Hebrew epoch by the mitre of the High Priest; the Greek epoch by the *Stephanos* or leafy crown worn by the victors in the games; the old Roman epoch by the civic crown; the Byzantine empire by the diadem; the middle ages by the tiara of the pope; the French revolution by the *bonnet rouge*, and more forceful still, an intelligent, cultured, refined community by the silk hat and the lawless, careless, unrestrained people of the frontier and the Far West by the broad-brimmed slouch wide-awake.

One can readily see how apt these things are when they are presented to him, as well as the fact that some very marked remnants of the past, so far as head-gear is concerned, have come down to us, although the reason for them may not be so apparent. One, as an instance, the custom or habit of uncovering the head by way of salutation or obeisance, a token of homage or deference or respect or courtesy which, in the depth and force of its meaning, is surpassed by no other demonstration that our civilization permits or ordains between man and man. It is a habit or custom whose origin goes very far back in the history of the world, having been originally the admission of an inferior to a superior of the power and strength of the latter, saying silently in substance: "See! I am uncovered. My head is yours as is all else that I have!"

It hardly means that much these days, except perhaps in Persia, Russia, and Japan.

It will be found that in all despotic countries some sort of head coverings have been worn, ever since there were heads to wear them, and mainly that this mark of deference and submissiveness might be observed. Ancient kings and rulers are always represented covered as to their heads, while those in their presence are bare-headed. A very ancient account mentions hats. This is made by Daniel whose other name has not come down to us, through whose writings, we are introduced to Nebuchadnezzar and some of his curious and rather inexcusable acts, which is far enough back in the past to go. He is telling the story of the three men whom the king tried but vainly to cremate. "They were bound," he says, "in their coats, their hosen, and their *hats*, and their other garments, and were cast into the midst of the fiery furnace."

One might be curious to know why they did not remove their hats at such a time, and on this inquiry some, of the faith of George Fox and William Penn, claim that these salamanders, in that respect, were among the original Quakers, or at least deserve to be so considered. It would hardly be worth while to controvert the statement, as perhaps it is due to this, that among those of the faith I have named there are so many that answer to the name either of Shadrach, Mesheck, or Abednego.

On the other hand where there were free races or nations or any pretence to liberty or equality, no covering was worn on the head, except when protection was called for from the weather or from enemies. In the early and free ages of the Hebrews, Parthians, Grecians, and Romans, the head was always bare, except in bad weather, in battle or during a long journey. Our own ancestors, the Germans, Saxons, and Celts, people as free as the air, scorned coverings for their head, their plentiful tawny locks being so thick and heavy that they were more or less a protection from the elements and even a shield against a good, stout blow from a club or spear. It was all an intimation that there were no superiors or inferiors among the nations or races named. All were uncovered and so all equal.

It might be worth while to inquire here, from another point of view, if the keeping of their heads covered by the English House of Commons while in session and of Hebrews while worshipping in their synagogues, arises from a notion that they are the governing forces and therefore are entitled to remain covered while in the exercise of their duties?

With the coming-in of the Cæsars and the departure of liberty from among the Romans, head-gear came generally into use. Julius Cæsar himself began it, as he began many an observance and custom that we, nearly two thousand years after him, are obediently following. He constantly wore a covering for his head, a simple one to be sure, but still a covering, it being only a wreath or crown of laurel leaves, the front projecting in a point several inches above his head. He himself hath said it that he wore this because he was very bald, and wanted to conceal the loss of his hair, which was considered a great deformity. This may have been so, but it likewise gave him the advantage of being always covered, while every one else, in his presence, was bare-headed, unmistakable marks of superiority on one side and of inferiority on the other.

There is a curious illustration of a certain head-gear of this period of the world that merits attention. One class of citizens wore a certain peculiar cap to distinguish them. These were the freedmen. When slaves were manumitted or given their liberty, they were permitted or perhaps commanded to assume a cap to distinguish them from others. This cap is precisely the one to which we now give the name of "liberty cap," a head covering familiar to us as always seen on the head of the figures of the Goddess of Liberty on our coins, our flags, and in newspaper illustrations. It is a loose sort of a garment like a pointed bag. It also curiously survives in the soft, drab hat worn by clowns in circuses, and is one of the appointments of the ideal figures of "Folly," or of a "Fool," or "Jester." This is not very good company for Liberty to be connected with, and her cap has no more savory origin, the curious part of it being that it can be traced back, with no steps wanting, to the covenant made with Abraham as set forth in Gen. xvii: 9-11.

For fourteen hundred years after Cæsar's time, men struggled along with many curious contrivances with which to cover and protect their heads, for no one save the very poor went bareheaded in those despotic times. There was a great deal of hard hitting in that period, and the head needed a tough and impervious covering, therefore most of the head-pieces were helmets of iron or steel.

It was the century of Columbus, Luther, Galileo, Guttenberg, and the other great men whom the world delights to honor, that also saw the emancipation of the head from its rude aud uncouth coverings and brought forth the hat substantially as we know it now. There was no evolution about it, no gradual growing up to it. It was an inspiration, a revelation, a creation. The name hat was given it, but this marked no change in sense if it did in orthography, for hat and cap mean precisely the same thing and come from precisely the same root: *chad*, to cover, a word in the old Sanskirt tongue, that language which lies at the foundation of nearly, if not quite all of our speech.

A Swiss invented the hat in 1404. He was living in Paris at the time, but his name has not been preserved. He deserves an immortality quite as much as does Kossuth, of whom it might be said that: "He could not give Hungary liberty, but he gave to America the slouched hat." Perhaps the matter is even however, for Kossuth begged a lot of money from us and lived on it, and the Swiss made a great fortune by the manufacture of his hats which became very fashionable, he religiously preserving until on his death-bed, the secret of his work. One of the kings of France wore a white felt hat of his make, in a royal procession about 1440, and set great store by it. Think of the Prince of Wales in his progress through Ireland completing his royal attire with a white felt hat!

The warriors of the fifteenth century took more than kindly to the new invention. They had become wonted to the stiff, hard griping of their steel and iron helmets, and the hat was not so much of a change, except in weight, that it could bear an unfavorable comparison.

Besides this the church treated the new article of wearing apparel much more graciously than it did the other inventions and discoveries of the era, like printing and the theory of the revolution of the earth about the sun. These latter she could by no means stomach, but the hat could readily be seen into and understood. Popes, cardinals, and bishops took to the hat as perhaps a relief from the other more exciting events and topics of the time. At the best, everything was hollow, the hat among them, and it was probably their influence that sent it, rolling and tumbling a long ways toward the immortality it seems likely to enjoy. One Pope couldn't think of a more striking evidence of his gratification and confidence than to bless a hat in solemn conclave and send it to a prince or commander who deserved well of the Roman Catholic Church. Perhaps from this custom came the expression, familiar in after years when a person has got the better in any sense of another, for the latter to say humbly: "Here. You may take my hat!"

For near five hundred years now this Swiss invention has held the masculine world subject to its iron-bound grasp; an example of perpetuity in a garment without parallel in the history of clothes among civilized people. Men may come and men may go, but the hat goes on forever. You may approve as much as you like of the soft, comfortable feeling of a Kossuth or sombrero, but no man can consider himself completely dressed who has not upon his head a regulation hat, smooth, glossy, and hard.

We have high authority on both sides, however as to whether comfort or style should rule as to the hat. "'Ta'nt a werry good un to look at," said Mr. Samuel Weller, as down he sat, without further bidding, having previously deposited his old white hat on the landing outside, "but it's an astonishin' 'un to wear ; and afore the brim went it was a werry handsome tile. Howsoever, it's lighter without it, that's one thing, and every hole lets in some air, that's another—wentilation gossamer, I calls it."

On the other hand Oliver Wendell Holmes, thus:

> "Have a good hat; the secret of your looks
> Lives with the beaver in Canadian brooks;
> Virtue may flourish in an old cravat,
> But man and nature scorn the shocking hat.
> Does beauty slight you from her gay abodes,
> Like bright Apollo, you must take to *Rhoades*,
> Mount the new castor—ice itself will melt;
> Boots, gloves may fail; the hat is always felt."

And further, George William Curtis:

"The hat is progress, liberality, civilization. The slouch is retroaction, barbarism, and chaos come again. Do the people who present themselves to public view in such things really mean what their hats say ? * * * * Let them remember that the lower, the limberer and plumier the hats of the cavaliers became, the higher and stiffer rose those of the Puritans. What occult sympathy is there between limpness of hats and looseness of principles ? Are these slouched hatters conspiring for the return of full hose and slashed doublets ? Are we to be plunged backward into the Roman toga ? Hold, hold your hats! Let us pause while there is yet time, and be content with the nineteenth century, happiness, and hats."

